# The thionin family of antimicrobial peptides

**DOI:** 10.1371/journal.pone.0254549

**Published:** 2021-07-14

**Authors:** Katharina Höng, Tina Austerlitz, Timo Bohlmann, Holger Bohlmann

**Affiliations:** Department of Crop Sciences, Institute of Plant Protection, University of Natural Resources and Life Sciences, Vienna, Austria; University of Agriculture Faisalabad, PAKISTAN

## Abstract

Thionins are antimicrobial peptides found only in plants. They are first produced as preproproteins and then processed to yield the usually 5 kDa, basic thionin peptide with three or four disulfide bridges. So far, thionins had only been found in some plant families of angiosperms. The One Thousand Plant Transcriptomes Initiative (1KP project) has sequenced the transcriptomes of more than 1000 plant species. We have used these data to search for new thionin sequences which gave 225 hits. After removing doublets these resulted in 133 new thionins. No sequences were found in algae and mosses. The phylogenetically earliest hits were from *Selaginella* species and from conifers. Many hits were from angiosperm plant families which were previously not known to contain thionins. A large gene family for thionins was found in *Papaver*. We isolated a genomic clone from *Papaver somniferum* which confirmed the general genomic structure with two small introns within the acidic domain. We also expressed the thionin encoded by the genomic clone and found that it had antimicrobial activity *in vitro*, especially against fungi. Previously, we had grouped thionins into four classes. The new data reported here led us to revise this classification. We now recognize only class 1 thionins with eight cysteine residues and class 2 thionins with six cysteine residues. The different variants that we found (and also previously known variants) can all be traced back to one of these two classes. Some of the variants had an uneven number of cysteine residues and it is not clear at the moment what that means for their threedimensional structure.

## Introduction

A large variety of relatively small, basic, and often cysteine-rich polypeptides have been isolated from different organisms and shown to have antimicrobial activities *in vitro* [1–3]. Some prominent examples from animals include magainins from the skin of *Xenopus laevis* and mammalian defensins and β-defensins. Antimicrobial peptides (AMPs) seem to be distributed ubiquitously in multicellular organisms. Plants have also been shown to contain a variety of different AMPs, including thionins [4,5] and plant defensins [6,7]. It is thought that the molecular targets of the majority of the usually basic AMPs are acidic phospholipids in biomembranes [8]. However, at least for some AMPs, biomembranes may just be a barrier hindering access to their primary target inside the cell [9–11].

**Fig 1:**
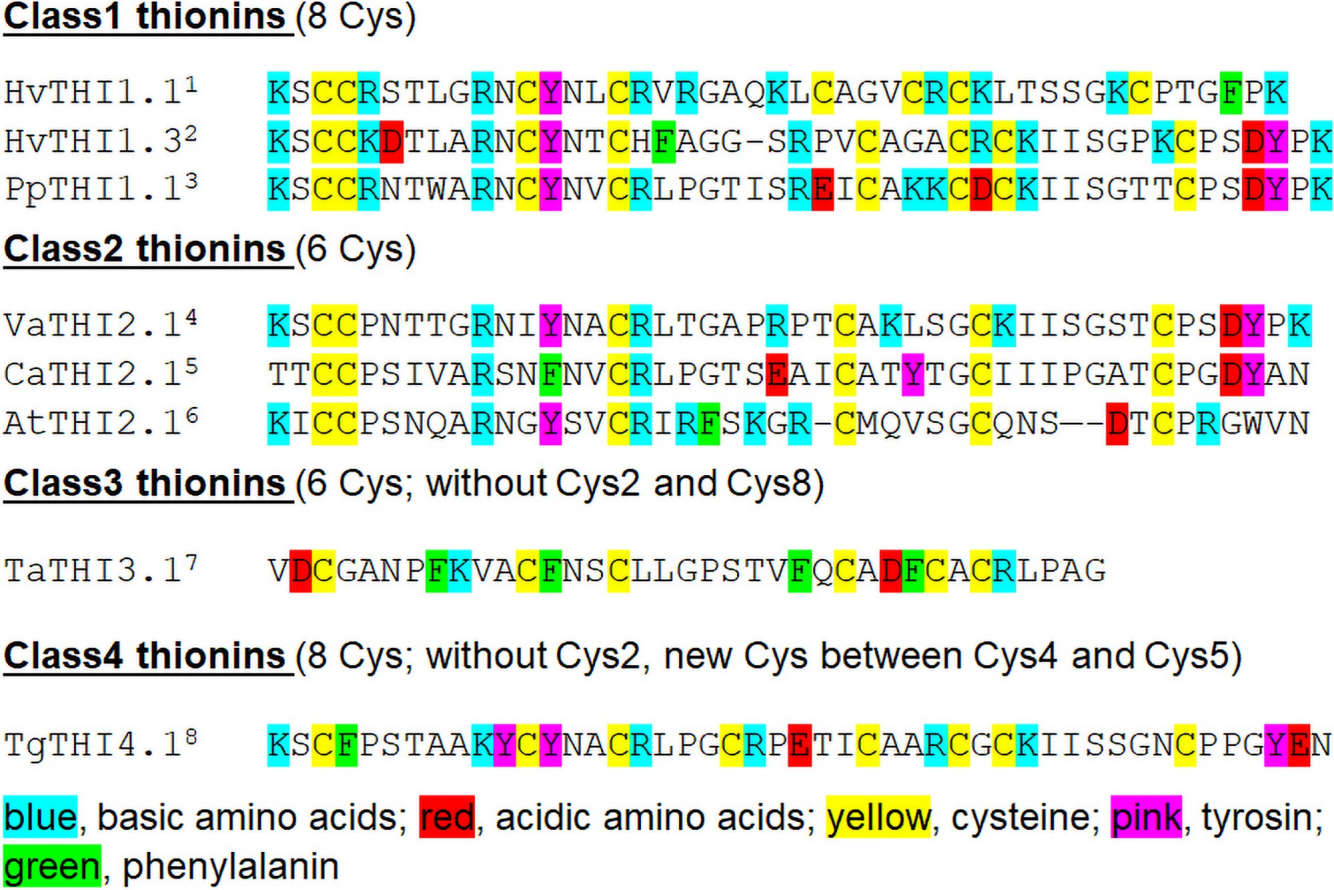
Examples of thionins including previous classification of thionins [18]. ^1^HTH1, Hordothionin α [19]; ^2^Barley leaf-thionin DB4 [20]; ^3^Pyrularia thionin [21]; ^4^Viscotoxin A3 [22,23]; ^5^Crambin 2 [24,25]; ^6^*Arabidopsis thaliana* thionin 2.1 [26]; ^7^pTTH20 Neutral wheat thionin [27]; ^8^Tulipa gesneriana thionin4.1 (Genbank, submitted by Luyten et al. 1994).

Thionins (Fig 1) are a group of AMPs whose toxic activity was apparently first described by Jago and Jago in 1885 [12] who reported that wheat flour contains a substance that was toxic to yeast. Balls and Hale [13] found that the polypeptide responsible for the toxicity could be extracted with petroleum ether and named it purothionin. It was later shown to consist of three different isoforms [14–16] whose sequences have been determined. Purothionins, as the majority of thionins, are basic and cysteine-rich polypeptides with a molecular weight of about 5 kDa. Related polypeptides, all with eight conserved cysteine residues, have been isolated and sequenced from the endosperm of oat and barley and are also present in other species of the *Poaceae* [17].

Cultivated barley (*Hordeum vulgare* L.) and also many wild *Hordeum* species contain a large multigene family of closely related genes for leaf thionins [20,28,29] in addition to the small gene family for endosperm specific hordothionins [19]. Mature leaf thionins have been found in the vacuole [30] and in the cell wall [28] of barley leaves. A prominent feature of leaf thionin genes in barley is their light responsiveness. Etiolated barley seedlings contain a very high level of leaf thionin transcripts which drops drastically after illumination, probably mediated by two photoreceptors, phytochrome and a blue-light-absorbing photoreceptor [31]. Barley leaf thionins and hordothionins have repeatedly been shown to have antimicrobial activities *in vitro* against different phytopathogenic bacteria and fungi [28,32,33]. This *in vitro* toxicity and the cellular distribution of barley leaf thionins suggested a role in plant defense.

Thionins are also present in dicot species. One large group of thionins (Fig 1) has been found in different species of mistletoes [17]. The toxic effects of viscotoxins towards mammals have been investigated in detail, for instance by Rosell and Samuelsson [34] because of the medical use of mistletoe extracts in the treatment of cancer [35]. Several thionins with six cysteine residues have been described from species of the family *Brassicaceae* (Fig 1). These include crambin with six cysteine residues from the seeds of *Crambe abyssinica* [36]. Crambin has a pI of 5.73 and has no known toxic activities [37]. Schrader-Fischer and Apel found in addition to crambin several novel and highly variable thionins in *Crambe abyssinica* using a PCR based approach [38]. Arabidopsis contains four thionin genes, of which two have been studied in detail [26,39–42], giving strong support for a role in plant defense.

Thionins are characterized by two consecutive cysteine residues at position 3 and 4. Some variants are known that are missing one of these cysteine residues. Accordingly, thionins have been classified [18] as class 1 with eight cysteine residues, class two with six cysteine residues and variants of class 3 and 4 (Fig 1).

The seed specific crambin has been used intensively for threedimensional structure analysis [43]. Structures have also been determined for several other thionins. The general threedimensional structure of thionins shows a compact molecule consisting of two alpha helices and two beta sheets (reviewed by [44]). The threedimensional structure is related to the toxicity and antimicrobial activity of thionins. Many studies have shown that thionins can induce cell membrane permeability [5]. A phospholipid binding site, which includes the tyrosine at position 13 (Fig 1) which has been shown to be indispensable for toxiciy, is present in all known structures of thionins with antimicrobial activity and it has been proposed that thionins interact with negatively charged phospholipids present in the cell membrane. This binding and the withdrawal of phospholipids disturbs the fluidity of the membrane and finally results in membrane lysis [44]. Another possible mechanism has been reported, claiming that thionins which are inserted into the membrane act as water channels. Water is delivered through these channels to the lipophilic center of the biomembrane, leading to local membrane disruption [45].

Thionins have been isolated as the 5 kDa mature peptides (Fig 1) from different plant species but are synthesized as much larger precursors [46]. Cloning of different thionin cDNAs and genomic clones confirmed the original observation and revealed that these mature thionins are first synthesized as preproproteins (S1 and S2 Figs). All precursors have a typical N-terminal signal peptide with a conserved signal peptide cleavage site that directs the proprotein into the endoplasmic reticulum. The proprotein consists of the thionin itself and a C-terminal acidic domain. In those cases where genomic DNA sequences are known [19,42,47], there are two small introns in the acidic domain (S2 Fig).

The function of the acidic domain is still not clear. Acidic domains have six cysteine residues (S1 Fig) which are highly conserved as are the cysteine residues in the thionin and are usually acidic (as the name says). An exception are the acidic domains deduced from the *Tulipa* thionin precursors that have only three cysteine residues (sequences submitted to GenBank by Luyten et al. 1994). In the case of the acidic crambin, the acidic domain too is slightly acidic (S1 Table). The acidic domain of the Arabidopsis THI2.3 proprotein is actually basic (S1 Table). A protein corresponding to the acidic domain has, to our knowledge, never been isolated from plants. To date, there is no experimental information available about possible functions of the acidic domain. But it is clearly not dispensable as shown by the high conservation of the cysteine residues, even in the case of viscotoxin precursors which have several deletions in the acidic domain [23]. Furthermore, Florack et al. [48] found that expression of α-hordothionin in transgenic tobacco plants without the acidic domain resulted in significantly lower levels of the mature thionin.

One possible function might be that the acidic domain contains information to guide the thionin through the secretory pathway to its final destination (vacuoles, cell walls, protein bodies). Another function of the acidic domain might be to neutralise the basic thionin and thereby protect the cell against its own toxin. A toxic effect of barley leaf thionins has been demonstrated against tobacco protoplasts and against barley protoplasts [49].

We have recently purified a thionin precursor processing enzyme (TPPE) from etiolated barley seedlings which contain large amounts of leaf-specific thionins [31]. We used a fluorescently labelled peptide that incorporated the flanking sequence between barley leaf-specific thionins and their acidic domains to identify a TPPE protein as the subtilase BAJ93208. The barley TPPE was produced as a *strep*-tagged protein in *E*. *coli* and used to study the processing of a recombinant leaf-thionin precursor *in vitro*. It could be shown that this protease produced thionins with a correct C-terminus while the acidic domain was cleaved several times. This explains why a peptide corresponding to the acidic domain has never been isolated from plants. Furthermore, it could be shown that the thionin domain was protected by its threedimensional structure against cleavage by the TPPE [50].

While plant defensins are ubiquitously distributed in plants, thionins were only found in a limited number of plant families. The One Thousand Plant Transcriptomes Initiative (1KP project) which sequenced the transcriptomes of more than 1000 plant species [51] was therefore a good opportunity to study the distribution of thionins in the plant kingdom. We searched the data obtained in that project for thionin sequences. We found thionin sequences from several families which had previously no reported thionins, including a large gene family in the genus *Papaver*. However, a large number of plants was without thionin sequences. We also found a number of sequences that would code for unknown thionin variants which prompted us to propose a new classification system for thionins. A genomic clone was isolated from *P*. *somniferum* and the encoded thionin was expressed in *E*. *coli* and shown to have antimicrobial activity.

## Materials and methods

### Database searches

We used BLAST to search the translated 1KP data with thionin amino acid sequences. As bait sequences we used a range of well-known thionins, for instance THI2.1 from Arabidopsis, VaTHI2.1 (Viscotoxin A3), CaTHI2.1 (Crambin), HvTHI1.3 (leaf thionin DB4 from Barley) and PpTHI1.1 (Pyrularia thionin) as well as some sequences that were found using the former baits (S2 Table). Searching was done with each of these bait sequences against all samples separately. From these results we removed the sequences which were repeatedly found by the different bait sequences. This resulted in a list of 2130 primary hits. All hits were then curated manually to remove the non-thionin sequences. Typically, thionins would have a double cysteine motif at position 3 and 4 as well as an additional four or six cysteines and an aromatic amino acid at position 13. Furthermore, we also looked for a possible acidic domain extension with its typical three and three cysteines. Manual inspection also allowed us to discover several thionin variants with untypical cysteine motifs. This produced a final list of 225 hits of which some resulted in the same sequences because the 1KP project often sequenced several different tissues for one species. Sometimes these different tissues were in addition pooled and sequenced, also resulting in redundancy. Signal peptides and acidic domain sequences were removed because they were often truncated. In some cases where the end of the signal peptide was not obvious (in case of typical thionin sequences with a basic amino acid at position 1) SignalP 5.0 (http://www.cbs.dtu.dk/services/SignalP/) was used to predict the signal peptide cleavage site [52]. The cleavage site between thionin domain and acidic domain was predicted according to known thionin sequences being six amino acids after the last cysteine.

Mw and pI were computed at https://web.expasy.org/compute_pi/. Pairwise alignment was performed by using emboss needle (https://www.ebi.ac.uk/Tools/psa/emboss_needle/) [53] and clustal omega (https://www.ebi.ac.uk/Tools/msa/clustalo/) was used for multiple sequence alignments [53]. Sequence logos were created at http://weblogo.threeplusone.com/create.cgi [54]. For creation of the logo two truncated class 1 sequences were removed. For the class 2 logo we removed a truncated sequence and two sequences (ChTHI2.1 and MfTHI2-1) with insertions in the middle for easier comparison with class 1.

### Cloning

All primers used for cloning can be found in S3 Table. Genomic DNA was prepared from *P*. *somniferum* seedlings according to [55]. A genomic clone was amplified using primers PsoThi1.5forNco and PsoThi1.5revBam. A fragment of approximately 730 bp was obtained. It was digested with NcoI and BamHI and ligated to the vector fragment of pMAAred [56] digested with the same enzymes, thus replacing the GUS sequence. The insert was sequenced and shown to be a thionin preproprotein with two introns.

For expression of the thionin encoded by the genomic clone we constructed an expression vector for *E*. *coli* which was based on a modified pETtrx1a vector [57] with an NdeI site at the start codon and a BamHI site behind the stop codon. The vector contained a His-tag in front of the thioredoxin sequence followed by a TEV site. We amplified the thioredoxin part of the vector by PCR using primers pETtrxfor1 and pETtrxTEVrev. The thionin coding sequence of the PsoTHI1.7 genomic clone was also amplified by PCR using the primers TEV1PsoTH1.7for and PsoTH1.7Bamrev. The thioredoxin fusion fragment and the thionin fragment were combined and amplified with primers pETtrxfor1 and PsoTH1.7Bamrev. The final PCR fragment was digested with NdeI and BamHI and cloned into the modified pETtrx1a vector previously digested with the same enzymes. The final expression vector was verified by sequencing.

### Expression and purification of PsoTHI1.7

The recombinant pETtrx::PsoTHI1.7 vector was transformed into the *E*. *coli* strain C3030 Shuffle [58]. A 100 ml TB medium pre-culture (containing 50 mg/ml kanamycin) was inoculated with a single colony and incubated overnight at 37°C and 180 rpm. The next day, 2 L TB medium (containing 50 mg/ml kanamycin) were inoculated with 3% v/v pre-culture and grown at 30°C and 140 rpm until the OD_600_ reached 1. Expression was induced by addition of 0.75 mM IPTG and incubated overnight at 20°C and 140 rpm. Cells were harvested by centrifugation for 30 min at 4°C (Sorvall RC6+ centrifuge, Thermo Scientific) and resuspended in 30 ml of ice-cold buffer (20 mM NaH_2_PO_4_, 0.5 M NaCl, 50 mM imidazole, pH 7.4). Cells were lysed by sonication (Sonifier W-250D, Branson Ultrasonics) in an ice-water bath. The lysate was clarified by centrifugation at 18,000 rpm for 30 min at 4°C.

The supernatant was used to isolate the fusion protein by immobilized metal ion affinity chromatography on an ÄKTA purifier10 FPLC system (GE healthcare) with a HisTrap HP 5ml column (GE Healthcare), equilibrated with 10 CV binding buffer (20 mM NaH_2_PO_4_, 0.5 M NaCl, 50 mM imidazole, pH 7.4). The sample was loaded with the sample pump at a flow rate of 5 ml/min and the column was washed with binding buffer until the 280 nm absorbance reached a steady baseline. The fusion protein was eluted with 2 CV of Elution buffer (20mM NaH_2_PO_4_, 0.5 M NaCl, 500 mM imidazole, pH 7.4) and collected with a fraction collector. Flow-through and the eluted fractions were analyzed by SDS-PAGE [59] using a Mini PROTEAN Tetra Cell 1mm (BioRAD). Fractions containing fusion protein were pooled and dialysed (SpectraPor RC tubing, MWCO 3.5 kDa Spectrumlabs) to a buffer containing 25 mM Tris-HCl pH 8, 0.5 mM EDTA, and 0.25 mM DTT was added directly to the sample before proteolytic digest. The fusion protein was cleaved using TEV protease at a ratio of 1:10 and cleavage was confirmed by PAGE (S3A Fig).

The cleaved fusion protein was dialysed to a binding buffer (20 mM NaH_2_PO_4_, 0.5 M NaCl, 5 mM imidazole, pH 7.4) for negative His-tag purification. Binding and loading to a column were performed as described before. The flow through, which contained PsoTHI1.7, was collected and the washing step was omitted. (His)6-TRX, remaining undigested fusion protein and the TEV protease were eluted (20 mM NaH_2_PO_4_, 0.5 M NaCl, 500 mM imidazole, pH 7.4) and discarded. The volume of the flowthrough was reduced to 500 μl using an Amicon Ultra centrifugal filter unit (MWCO 3 kDa). The sample was applied to a Superdex 75 10/300 GL column on an ÄKTA purifier10 (GE Healthcare) using the 100 μl sample loop with a flow of 0.5 ml/min. The running buffer was 100 mM TRIS-HCl pH 8, 500 mM NaCl. The purified peptide was run on a PAGE gel (S3B Fig) and silver stained according to [60]. For protein quantification, Thermo Scientific^™^ Pierce^™^ BCA Protein Assay kit was used. Page Ruler Unstained (Thermofisher 26614) and Page Ruler Unstained Low range (Thermofisher 26632) were used as PAGE marker.

### Antimicrobial assays

Antimicrobial assays were done according to [61]. Fungal test organisms were *Botrytis cinerea* 05.10 and *Fusarium oxysporum* f.sp. *matthiolae*. Both fungi were grown on PDA for two weeks at room temperature. *B*. *cinerea* was grown in the dark while *F*. *oxysporum* was grown in the light on the lab bench. Spores were harvested by flooding the plate with 10 ml of sterile water and filtering the spore suspension through two layers of cheesecloth. The spores were counted in a Thoma hemocytometer and the number of spores per μl was calculated. Spore concentration was adjusted to 2 x 10^4^ spores/ml in ½ concentrated PDB. 25 μl of the test peptide were pipetted into the wells of a clear 96 well cell culture microplate (PS,TC, F-bottom with a lid, Greiner Bio-One). Then 75 μl of the spore suspension was added. Final peptide concentrations were 100 μg/ml, 50 μg/ml, 25 μg/ml, 12.5 μg/ml, 6.25 μg/ml, 3.125 μg/ml, 1.56 μg/ml and 0 μg/ml. Plates were incubated in the dark at room temperature. The OD_600_ was measured on a Fluostar Omega Plate reader (BMG Labtech) using the well scan setting with a diameter of 3 mm and nine measuring points and double orbital shaking for 30 sec. before the measurement. OD was measured at the beginning of the experiment and again after 24 h and 48 h. The percentage of growth inhibition was calculated, using the median of all measuring points per well, as follows:

$$\%\;\text{growth}\;\text{inhibition}=100\;\text{x}\;\left(\text{absorbance}\;\text{of}\;\text{control}–\text{absorbance}\;\text{of}\;\text{test}\right)/\text{absorbance}\;\text{of}\;\text{control}$$

Bacteria were tested in a similar way. Test organisms were *Agrobacterium tumefaciens* GV3101 and *Pseudomonas syringae* pv. *tomato* DC3000. Bacteria were grown in 5 ml LB medium over night at 30°C. The cultures were centrifuged for 10 min at 7000 rpm and resuspended in 1% tryptone. Bacteria were diluted to an OD_600_ of 0.05 in 1% tryptone before using them for the test. The peptide was pipetted into a 96 well plate as described above and 75 μl of bacterial culture was added. OD_600_ was measured in a Fluostar Omega plate reader as described above. Plates were incubated in the dark at 30°C and measured again after 24h. Percentage of growth inhibition was calculated as described above.

## Results

### New thionins discovered by the 1KP project

The 1KP project sequenced the transcriptomes of more than 1000 plant species including algae, mosses, ferns, gymnosperms and angiosperms [51,62,63]. We used these data to search for thionin sequences in the translated protein sequences. Our search produced 225 hits (Table 1) of which some resulted in the same sequences because the 1KP project often sequenced different tissues from the same species. We thus ended up in a total of 133 thionin sequences (Table 2).

**Table 1:** Total hits obtained.

species/genus	family	order	Class I/II hits		
			I	II	both
*Selaginella wallacei*	*Selaginellaceae*	*Selaginellales*	1		1
*Selaginella stauntoniana*	*Selaginellaceae*	*Selaginellales*	1		1
*Cystopteris fragilis*	*Cystopteridaceae*	*Polypodiales*		2	2
*Manoao colensoi*	*Podocarpaceae*	*Coniferales*	1		1
*Sequoiadendron giganteum*	*Cupressaceae*	*Coniferales*	1		1
*Athrotaxis_cupressoides*	*Cupressaceae*	*Coniferales*	2		2
*Papaver*	*Papaveraceae*	*Ranunculales*	72	21	93
*Thalictrum_thalictroides*	*Ranunculaceae*	*Ranunculales*	1	2	3
*Hakea prostrata*	*Proteaceae*	*Proteales*		2	2
*Oresitrophe_rupifraga*	*Saxifragaceae*	*Saxifragales*	3		3
*Ribes_aff*.*_giraldii*	*Grossulariaceae*	*Saxifragales*	1		1
*Sesuvium_*	*Aizoaceae*	*Caryophyllales*		10	10
*Cypselea humifusum*	*Aizoaceae*	*Caryophyllales*		3	3
*Trianthema portulacastra*	*Aizoaceae*	*Caryophyllales*		6	6
*Zaleya pentandra*	*Aizoaceae*	*Caryophyllales*		1	1
*Aerva*	*Amaranthaceae*	*Caryophyllales*		8	8
*Alternanthera caracasana*	*Amaranthaceae*	*Caryophyllales*		2	2
*Polygonum convolvulus*	*Polygonaceae*	*Caryophyllales*	1		1
*Polycarpaea repens*	*Caryophyallaceae*	*Caryophyllales*		3	3
*Portulaca mauii*	*Portulacaceae*	*Caryophyllales*		2	2
*Mirabilis jalapa*	*Nyctaginaceae*	*Caryophyllales*		1	1
*Sassafras albidum*	*Lauraceae*	*Laurales*	1		1
*Lindera benzoin*	*Lauraceae*	*Laurales*	1		1
*Cannabis sativa*	*Cannabaceae*	*Rosales*		7	7
*Humulus lupulus*	*Cannabaceae*	*Rosales*		4	4
*Urtica dioica*	*Urticaceae*	*Rosales*		2	2
*Linum perenne*	*Linaceae*	*Malpighiales*		2	2
*Chrysobalanus icaco*	*Chrysobalanaceae*	*Malpighiales*	1		1
*Viola tricolor*	*Violaceae*	*Malpighiales*	1		1
*Brassica nigra*	*Brassicaceae*	*Brassicales*		3	3
*Arabis alpina*	*Brassicaceae*	*Brassicales*		6	6
*Sinapis alba*	*Brassicaceae*	*Brassicales*		3	3
*Draba*	*Brassicaceae*	*Brassicales*	3	6	9
*Cochlearea officinalis*	*Brassicaceae*	*Brassicales*		1	1
*Daphne geraldii*	*Thymelaeaceae*	*Malvales*	2		2
*Sarcodes sanguinea*	*Ericaceae*	*Ericales*	1		1
*Eleusine coracana*	*Poaceae*	*Poales*	4		4
*Panicum miliaceum*	*Poaceae*	*Poales*	1		1
*Neurachne*	*Poaceae*	*Poales*	3	3	6
*Thyridolepis*	*Poaceae*	*Poales*	2	2	4
*Lepidosperma gibsonii*	*Cyperaceae*	*Poales*		1	1
*Polyscias fruticosa*	*Araliaceae*	*Apiales*	1		1
*Psychotria ipecacuanha*	*Rubiaceae*	*Gentianales*		2	2
*Myristica fragrans*	*Myristicaceae*	*Magnoliales*		3	3
*Hydrocotyle umbellata*	*Araliaceae*	*Apiales*		1	1
*Prunella vulgaris*	*Lamiaceae*	*Lamiales*		1	1
*Teucrium chamaedrys*	*Lamiaceae*	*Lamiales*		1	1
*Orobanche fasciculata*	*Orobanchaceae*	*Lamiales*	3		3
*Schlegelia parasitica*	*Schlegeliaceae*	*Lamiales*	2		2
*Heliotropium texanum*	*Boraginaceae*	*Boraginales (Lamiales)*		2	2
*Phoradendron serotinum*	*Santalaceae*	*Santalales*		2	2
			110	115	
Total hits					225

**Table 2:** All thionin sequences from the 1 KP project.

name	Species	class	sequence	hits	pI	Mw (Da)
HvTHI1.1^1^	*Hordeum vulgare*	1	KSCCRSTLGRNCYNLCRVRGAQKLCAGVCRCKLTSSGKCPTGFPK	-	9.75	4855.81
CaTHI2.1^2^	*Crambe abyssinica*	2	TTCCPSIVARSNFNVCRLPGTSEAICATYTGCIIIPGATCPGDYAN	-	5.73	4726.42
VaTHI2.1^3^	*Viscum album*	2	KSCCPNTTGRNIYNACRLTGAPRPTCAKLSGCKIISGSTCPSDYPK	-	9.30	4835.60
AtTHI2.1	*Arabidopsis thaliana*	2	KICCPSNQARNGYSVCRIRFSKGRCMQVSGCQNSDTCPRGWVN	-	9.43	4811.52
TaTHI3.1^4^	*Triticum aestivum*	3	VDCGANPFKVACFNSCLLGPSTVFQCADFCACRLPAG	-	5.90	3826.47
TgTHI4.1	*Tulipa gesneriana*	4	KSCFPSTAAKYCYNACRLPGCRPETICAARCGCKIISSGNCPPGYDY	-	8.71	5040.85
SwTHI1.1^9^	*Selaginella wallacei*	1	KSCCPSTAARNCYNACRLVGTSQTTCASLCGCIHVDGNTCPPNYPS	1	8.21	4769.40
SsTHI1.1^6^	*Selaginella stauntoniana*	1	KSCCPSTAARNCYNACRLVGTSQTTCASLCGCIHVDGNTCPPNYP	1	-	-
McTHI1.1^10^	*Manoao colensoi*	1	KSCCPSTAARNCYNACRLVGTSQTTCASLCGCIHVDGNTCPPNYPK	1	8.51	4810.50
SgTHI1.1	*Sequoiadendron giganteum*	1	KSCCKNTTGRNCYNACRFAGGSRPVCATACGCKIISGPTCPRDYPK	1	9.25	4860.65
AcTHI1.1^10^	*Athrotaxis_cupressoides*	1	KSCCPSTAARNCYNACRLVGTSQTTCASLCGCIHVDGNTCPPNYPK	2	8.51	4810.50
OrTHI1.1^11^	*Oresitrophe_rupifraga*	1	KSCCVNTTARNCYNVCRLTGTQAFCANLCGCIHIDGTTCPPDYPK	1	8.21	4857.60
OrTHI1.2^6^	*Oresitrophe_rupifraga*	1	XSCCPSTAARNCYNACRLVGTSQTTCASLCGCIHVDGNTCPPNYPK	1	-	-
OrTHI1.3^6^	*Oresitrophe_rupifraga*	1	ARNCYNVCRLSGASRATCAKLCGCIHINGSTCPSNYPK	1	-	-
VtTHI1.1^9^	*Viola tricolor*	1	KSCCPSTAARNCYNACRLVGTSQTTCASLCGCIHVDGNTCPPNYPS	1	8.21	4769.40
SpTHI1.1^6^^,^^9^	*Schlegelia_parasitica*	1	KSCCPSTAARNCYNACRLVGTSQTTCASLCGCIHVDGNTCPPNYP	2	-	-
RgTHI1.1^6^	*Ribes giraldii*	1	TTARNCYNTCRLTGTSQARCASLCGCIHITGTTCPSN	1	-	-
DgTHI1.1	*Daphne geraldii*	1	KSCCRNTLGRNCYNTCRFGGAPRPVCASLCDCINIDGTRCPNTHPS	2	8.76	4976.68
SasTHI1.1^6^	*Sarcodes sanguinea*	1	KSCCKNTTGRNCYNACRFAGGSRPVCATACGCKIISGPTCP	1	-	-
PrThi1.1	*Papaver rhoeas*	1	RSCCDSKAGRNCYQACIRRTGATQLCARSCGCRFTRENRCPSSHPW	2	9.37	5197.91
PrThi1.2	*Papaver rhoeas*	1	KSCCNTTIKRNCYNICRFKFSQETCAKTCGCTLIHGTKCPNGNIV	1	9.08	5003.87
PrThi1.3	*Papaver rhoeas*	1	KSCCKTTIKRNCYNICRLKFSQETCAKTCGCTLIQGKKCPSRNDN	2	9.35	5091.00
PrThi1.4	*Papaver rhoeas*	1	KSCCRNTTARNCYNLCRVPGTPREVCAKACDCKIISGKKCPSDYPS	2	9.06	5041.88
PrThi1.5	*Papaver rhoeas*	1	RICCKDSVARSCFNSCQPGTPRSVCATTCRCRTISGLCPSSYPS	6	8.97	4703.43
PrThi1.6	*Papaver rhoeas*	1	KICCKSTTARSCFKACRIRLSRETCASTCSCKILTGNCPSDYPK	1	9.25	4835.72
PrThi1.7	*Papaver rhoeas*	1	KPCCTTYNGKKCNNRCRWDGGNKDSCADMCGCKSCPRIEVG	1	8.72	4503.17
PrThi1.8	*Papaver rhoeas*	1	KSCCKSTVGRNCYNACRLKFARQVCASTCSCKIVGGNRCPPGYPK	6	9.54	4859.75
PrThi1.9	*Papaver rhoeas*	1	KSCCKSTLGRNCYNACRLKFPRKTCSTCSCKILKGNRCPSGYPK	1	9.74	4905.86
PrThi1.10	*Papaver rhoeas*	1	KSCCKSTLGRNCYNACRLRLPRKTCASTCSCKILKGNRCPSGYPK	1	9.80	4970.94
PrThi1.11	*Papaver rhoeas*	1	KSCCKSTVGRNCYNACRLRFARQVCASTCSCKIVGGNKCPRGYPK	3	9.70	4918.82
PsoThi1.1	*Papaver somniferum*	1	RSCCGSKAGRNCYQACVRRTGATQLCARSCGCRFTRENRCPSSHPW	2	9.60	5125.85
PsoThi1.2	*Papaver somniferum*	1	KSCCKTTAARNCYNVCRLTGTSRQVCAATCGCKIISGNKCPRGYDK	5	9.38	4934.77
PsoThi1.3	*Papaver somniferum*	1	KNCCKTAFGRHCYNLCRLTSPRQNCDAICNCIRWGFSRCPRTYPH	1	9.30	5295.15
PsoThi1.4	*Papaver somniferum*	1	KSCCKSTVGRNCYNACRLRFARQVCASTCSCKIVGGNRCPRGYPK	1	9.75	4946.83
PsoThi1.5	*Papaver somniferum*	1	KSCCKST**I**GRNCYNACRL**R**FARQVCASTCSCKIVGGNRCPPGYPK	4	9.58	4901.79
PsoThi1.6^12^	*Papaver somniferum*	1	KSCCKSTVGRNCYNACRL**R**FARQVCASTCSCKIVGGNRCPPGYPK	6	9.58	4887.76
PsoThi1.7^13^	*Papaver somniferum*	1	KSCCKST**V**GRNCYNACRL**K**FARQVCASTCSCKIVGGNRCPPGYPK		9.54	4859.75
PseThi1.1	*Papaver setigerum*	1	KSCCKSTVGRNCYNACRLRFARQVCASTCSCKIVGGNRCPRGYPK	2	9.75	4946.83
PseThi1.2^12^	*Papaver setigerum*	1	KSCCKSTVGRNCYNACRLRFARQVCASTCSCKIVGGNRCPPGYPK	2	9.58	4887.76
PseThi1.3	*Papaver setigerum*	1	KSCCKSTLGRNCYNACRLRFPRKTCASTCSCKIVRGNRCPSGYSK	2	9.86	5008.90
PbThi1.1	*Papaver bracteatum*	1	RSCCDSKAGRNCYQACVTRTGATKLCARSCGCRFTRENRCPSSHPW	2	9.33	5128.84
PbThi1.2	*Papaver bracteatum*	1	KSCCQNTLARNCYNVCRFAGGSREACAKACNCKIITETDCPSDYPK	2	8.50	5037.76
PbThi1.3	*Papaver bracteatum*	1	KSCCKSASGRRCYNVCRLRFPRQNCGAICNCIAWWASNTCPYFAPY	1	9.27	5270.14
PbThi1.4	*Papaver bracteatum*	1	KSCCKSTFGRNCYNACRLKFPRKTCASTCSCKIVGGNRCPPGYPK	2	9.65	4935.85
PbThi1.5	*Papaver bracteatum*	1	KSCCKSTLGRNCYNACRLKFPRKTCASTCNCKILKGNKCPSGYPK	1	9.70	4975.95
PbThi1.6	*Papaver bracteatum*	1	KSCCKSTCYNACRLKFPRKTCASTCNCKILKGNKCPSGYPK	1	9.57	4535.45
PbThi1.7	*Papaver bracteatum*	1	KSCCKSTAARECYNACHSAGAPRYVCPYICKCLIISGTKCPPAYRY	7	9.03	5051.96
SaTHI1.1	*Sassafras albidum*	1	KSCCRSTTARNCYNVCRLSGSSRPTCASLCDCKIITGTTCPSDYPK	1	8.92	4952.69
LbTHI1.1	*Lindera benzoin*	1	KSCCRSTTARNCYNVCRLAGTPRETCAKLCDCIIITGTTCPSGYPK	1	8.92	4960.80
CiTHI1.1^11^	*Chrysobalanus icaco*	1	KSCCVNTTARNCYNVCRLTGTQAFCANLCGCIHIDGTTCPPDYPK	1	8.21	4857.60
PmTHI1.1	*Panicum miliaceum*	1	KSCCKSTLARNCYNVCRLRGARSVCATTCGCKIIKGTKCPPGYPK	1	9.65	4840.83
PfTHI1.1^11^	*Polyscias fruticosa*	1	KSCCVNTTARNCYNVCRLTGTQAFCANLCGCIHIDGTTCPPDYPK	1	8.21	4857.60
TtTHI1.1	*Thalictrum thalictroides*	1	KSCCPGTLQRNCYNLCRVGGKITSETCAKTCGCKHVVGRVCPPGWQS	1	9.10	5030.90
DsTHI1.1	*Draba sachalinensis*	1	KSCCPSTSARNCYNVCRVTGTSQRTCASLCGCKIISGNTCPPGFPS	3	8.95	4760.48
EcTHI1.1	*Eleusine coracana*	1	ISCCPDTTKRNCYNVCRHSMKKEICANVCGCKLVSGVKCPRDYPK	4	9.05	5053.01
NaTHI1.1	*Neurachne alopecuroidea*	1	KSCCRSTTARNCYNLCRLRRPQATCASLCGCKIIKGNTCPRDFPK	1	9.63	5038.97
NmTHI1.1	*Neurachne munroi*	1	KSCCKNTAGRNCYNICRRAGGSQQVCARRCGCIIITGNRCPPNYPK	1	9.63	5035.89
TmuTHI1.1^7^	*Thyridolepis multiculmis*	1	KSCCKSTMARNCYNICRFKGPRLVCAQMCGCKIIGGQKCPSDFPK	1	9.38	4976.03
TmiTHI1.1^7^	*Thyridolepis mitchelliana*	1	KSCCKSTMARNCYNICRFKGPRLVCAQMCGCKIIGGQKCPSDFPK	1	9.38	4976.03
OfTHI2.1	*Orobanche fasciculata*	1	KSCCEDTTARYCYNVCRLPGTPRQTCAKICGCIITTSTTCPSNYPK	3	8.72	5052.85
		**53**			**9.18**	**4939.41**
PiTHI2.1	*Psychotria ipecacuanha*	2	TTSCCPSAYARSTYNLCSLYKSQIICARLSGCILIDGTSCPSNYPK	2	8.62	4954.71
MfTHI2.1	*Myristica fragrans*	2	ESCCPSAKAKNLYNVCRNQYSDPHYFTKSFCANLAGCKLADGKKCEPPYDH	2	8.31	5717.46
CfTHI2.1	*Cystopteris fragilis*	2	KSCCPTIVARNQYSVCRFAGASRPECAKLSGCKIVDGECPGGYNR	1	8.90	4796.53
CfTHI2.2	*Cystopteris fragilis*	2	KTCCPSSTARSIYRTCRFGGSTQTCAQISGCKIVSGECPGGYNK	1	9.13	4608.25
HtTHI2.1^6^	*Heliotropium_texanum*	2	KSCCPSTTARNTYNVCRLAGTPRPVCASISGCKIITGTKCPKGY	2	-	-
MjTHI2.1	*Mirabilis jalapa*	2	KSCCPSTTARNIYNTCRFGGGSRPMCASISGCKIISGTKCPKGYEK	1	9.46	4863.67
HpTHI2.1^14^	*Hakea prostrata*	2	KVACCPSIAASNYYSICRLYGASGPKCAKIEDCKIVDGEECPGSTYP	2	6.24	4962.69
LgTHI2.1^14^	*Lepidosperma gibsonii*	2	KVACCPSIAASNYYSICRLYGASGPKCAKIEDCKIVDGEECPGSTYP	1	6.24	4962.69
TcTHI2.1	*Teucrium chamaedrys*	2	KSCCPSTSARNIYNTCRLAGGTRPFCASISGCKIVDGKCPTGWDK	1	9.13	4754.49
PrThi2.1	*Papaver rhoeas*	2	TSCCPSAYARSTYNLCSLYKSQIICARLSGCILIDGTSCPSNYPK	7	8.62	4853.61
PrThi2.2	*Papaver rhoeas*	2	KICCMNDTRRNRYKDCLNTGASVTSCAGVSGCLIVSGSLCPPNYPY	4	8.65	4932.69
PsoThi2.1^5^	*Papaver somniferum*	2	TSCCESTKARNSYSVCRLRLGASKNCAKLTGCIIIDGTSCPSDYPI	3	8.63	4887.63
PseThi2.1^5^	*Papaver setigerum*	2	TSCCESTKARNSYSVCRLRLGASKNCAKLTGCIIIDGTSCPSDYPI	3	8.63	4887.63
PbThi2.1	*Papaver bracteatum*	2	KICCMNDTRRNRYEVCLNTGASVASCAGVSGCLIISGSVCPPNYPH	4	8.34	4861.61
CoTHI2.1	*Cochlearea officinalis*	2	TLCCPNKKTADIYATCRTSGVSKYMCERLSGCKNVSGTCPDILQN	1	8.62	4874.65
HuTHI2.1	*Hydrocotyle umbellata*	2	KSCCPNTTARNIYNTCRITGASRSVCASLSGCIIQSSSTCLPPNTH	1	8.96	4818.48
PvTHI2.1	*Prunella vulgaris*	2	KSCCPSTSARNIYNVCRLPGTARETCAKLSGCKIQDPPCVPPFDH	1	8.66	4865.63
TtTHI2.1	*Thalictrum thalictroides*	2	KSCCPSTLKRNIYNACRLKFSQETCAKTSGCKLEDKTCPEGWQK	1	9.06	4985.78
TtTHI2.2	*Thalictrum thalictroides*	2	YVVCCKNIQARNYFNACLSLGSGTSDCLRHSNGNCIRKTGATCPANFPR	1	9.15	5326.08
SvTHI2.1	*Sesuvium ventricosum*	2	KSCCPSTTARNTYNVCRLAGTPRPMCASISGCKIITGTKCPKGYEK	1	9.46	4897.77
ChTHI2.1	*Cypselea humifusum*	2	KSCCPNTTARNIYNTCRSVQTPAEIYKACRITGGTRSFCAQLSGCKIKKLT	1	9.64	5587.53
ChTHI2.2^6^	*Cypselea humifusum*	2	LSCCPSTTSVRHVFSSCRLAGGSRSVCASLSGCKIVSGATCPRAYDK	1	9.18	4810.55
TpTHI2.1	*Trianthema portulacastrum*	2	KSCCPSTTARNTYNVCRLAGTPRPVCASISGCKIITGTKCPKGYEK	1	9.46	4865.71
TpTHI2.2	*Trianthema portulacastrum*	2	KSCCPNTTARNIYNTCRAAGGSRPQCASLSGCIHISGNKCPSTHPK	1	9.38	4792.45
TpTHI2.3	*Trianthema portulacastrum*	2	KSCCRSTTARNAYNVCRFAGGTQSQCATVSGCKIISGNKCPSDYPK	2	9.30	4863.53
TpTHI2.4	*Trianthema portulacastrum*	2	KSCCRSTTARNAYNVCRFAGGSQGQCATVSGCKIISGNKCPSDYPK	1	9.30	4819.47
ApTHI2.1	*Aerva persica*	2	KSCCPSTSARNIYNTCRFTGGSREMCASLSGCKIIKGTKCPPGYEK	2	9.27	4935.74
AcTHI2.1^6^	*Alternanthera caracasana*	2	XSCCPSTSARNIYNVCRLGGASREVCAKLSGCKHMDACKPPYIH	2	-	-
PrTHI2.1	*Polycarpaea repens*	2	KSCCPNTTARNIYNACRLTGASREFCANLSGCKILDVTTCPSDYPS	1	8.33	4946.61
PrTHI2.2	*Polycarpaea repens*	2	KSCCPTEKARISYMFCLKSEVNDQALCASASGCKIAKDRICPPGYDH	1	8.30	5139.96
CsTHI2.1	*Cannabis sativa*	2	KSCCPSTTARNIYNTCRFGGGSRETCASISGCRIITGNTCPGGWTN	5	8.96	4803.39
CsTHI2.2	*Cannabis sativa*	2	KSCCPSTTARNIYNTCRFGGGSRETCASISGCRIITGNTCPGGNDH	1	8.69	4768.30
CsTHI2.3	*Cannabis sativa*	2	KSCCPTTTARNIYNACRLTGDSRKFCASISGCKIISGNTCPDGYNH	1	8.90	4929.59
UdTHI2.1	*Urtica dioica*	2	KSCCPNTYARTSYDFCRSNHGPEIRCSLESGCLIVDNGARCPPDYPI	1	6.71	5210.85
UdTHI2.2	*Urtica dioica*	2	XSCCPTTTARKIYNTCRFAGGTRPFCASVSGCKIVTGKCPPHYDK	1	-	-
HlTHI2.1^6^^,^^8^	*Humulus lupulus*	2	KSCCPSNSARNNYNICRLGGIPQSTCAQLTGCQIISGS	2	-	-
HlTHI2.2^8^	*Humulus lupulus*	2	KSCCPNNSARTTFNVCRFGGASQSICASLSGCQLISGSTCPSGFTH	2	8.70	4687.26
LpTHI2.1	*Linum perenne*	2	KSCCPTTAARNIYDACRFAGGSREFCAKLSGCKIISGNTCPDGYNH	2	8.65	4888.54
BnTHI2.1	*Brassica nigra*	2	TSCCPSSSARNTYNVCRLPGTPRPICASISGCKIVSGTCPPGYAN	1	8.93	4591.26
BnTHI2.2	*Brassica nigra*	2	MICCPTARARYLFDVCSSRRISIIICSGTTGCVPIPGDTCPPDYPG	1	7.56	4907.76
AaTHI2.1	*Arabis alpina*	2	KSCCPSTNARNVYNLCLLVQHPRSVCARISQCKIVQENTCPPGYPK	1	9.13	5120.99
AaTHI2.2	*Arabis alpina*	2	FACCPTDAARDIFSECTYSGKVWTFCASLSGCQLNRYHNCPPGYTH	1	6.87	5122.75
AaTHI2.3	*Arabis alpina*	2	WTCCPTDAARDMYFECTYSGKDPTDCALLSDCKLDIWGDCPPGYDH	1	4.04	5180.75
AaTHI2.4	*Arabis alpina*	2	ATICCPTTKARTAYNFCRLRGTSSLICAYRNGCKINEEDECPPDFPK	1	8.33	5259.03
AaTHI2.5	*Arabis alpina*	2	IQACCPTREAHKIFDKCIGGYKGYIRCASVSGCLIIEENNCPPGFTH	1	7.78	5144.97
AaTHI2.6	*Arabis alpina*	2	AVCCPDRYARNFYNFCLQLGEIYLPTCLSKTGCQIFPMCPKDTDD	1	4.78	5154.97
SaTHI2.1	*Sinapis alba*	2	KSCCPSITARNTYNVCRFTGSSRQTCARLSGCTIVSGTTCPQGYPH	1	9.18	4914.57
SaTHI2.2	*Sinapis alba*	2	RVCCPNNLDRKKFDLCSATGFPKSACARLSECKIFPGNTCPSDFPA	1	8.66	5009.80
SaTHI2.3	*Sinapis alba*	2	KTCCPTTNARHIIDRCLAIGIPMIICLDTTGCVISTRPTCPPNFPA	1	8.35	4929.90
DsTHI2.1	*Draba sachalinensis*	2	KTCCRTAVARNIYNRCRRTGGSIVFCVQSSRGLCRIFPGGRPCRPPFSF	1	11.17	5496.48
DhTHI2.1	*Draba hispida*	2	RTCCLTVADRDIFSECTYSGKIWTFCASLSGCKLMPRYSCPPGYIH	1	8.32	5182.04
DoTHI2.1	*Draba oligosperma*	2	KYCCVSTNARKNFNTCLSKGIPSTTCAYDSGCKFTYTNTCPPGYAD	1	8.62	4996.62
DaTHI2.1	*Draba aizoides*	2	KSCCPSTSARNIYNTCRFAGGSRPTCASVSGCKIVSGTCPPGFTH	1	9.18	4611.26
DmTHI2.2^6^^,^^8^	*Draba magellanica*	2	KSKKACCPSYDARNVYRICRIRLNQPDCASLSECILIDNGNCPAG	2	-	-
NlTHI2.1	*Neurachne lanigera*	2	NVCCPSKLTRTVYNRCYRLVGSETECAEWTTCKVVDGECKPPYDH	1	6.72	5155.86
NaTHI2.1^14^	*Neurachne alopecuroidea*	2	KVACCPSIAASNYYSICRLYGASGPKCAKIEDCKIVDGEECPGSTYP	1	6.24	4962.69
TmiTHI2.1	*Thyridolepis mitchelliana*	2	KSCCPSTTARNIYNVCRLAGRSRAVCAKLSGCKIVGTGEKCPDDYIH	1	9.10	5046.87
TmuTHI2.1	*Thyridolepis multiculmis*	2	KSCCPSTTARNIYNACRFAGSSRPVCAKLSGCKIVGTGEKCPDSHIH	1	9.13	4955.71
MfTHI2.2	*Myristica fragrans*	2	KSCCPSTTARNIYNTCRVPGTARPVCAKLSGCIIQEAKKCEPPYDH	1	8.89	5011.82
PsTHI2.1	*Phoradendron serotinum*	2	KVCCPTTAARNMYDPCRATPPICARNAGCIIILGSKCPRGWDH	1	8.94	4661.51
PsTHI2.2	*Phoradendron serotinum*	2	KVCCPSTAARNIYNACRARPPGCAYAAGCIIILGSNCPGGWDH	1	8.68	4452.16
PrTHI2.3^6^	*Polycarpaea repens*	2	XXXXXXXXXRNIYNACRLAGGSRERCASLAGCKIVSGTTCPSSLPN	1	-	-
		**62**			**8.47**	**4959.49**
PrTHI-BEKN-18329*	*Papaver rhoeas*	1X	KSCCKSTVGRNCYNACRLKFARTCSCKIVGGNKCPRGYPK	1	9.78	4402.25
PsoTHI-RQNK-5714*	*Papaver somniferum*	1X	KSCCYNACRLRFARKVCASTCSCKIVGGNRCPRGYPK	1	9.73	4100.91
PseTHI-JSVC-2507	*Papaver setigerum*	1X	KSCCKSTVGRNVGYARQVCASTCSCKIVGGNRCPRGYPK	1	9.70	4138.85
PseTHI-QCOU-26075*	*Papaver setigerum*	1X	KSCCKSTVGRNCYNACRLRFARQVCASTCSCKIVGGNRCKIVGGNRCPRGYPK	1	9.88	5774.83
PbTHI-SSDU-32671*	*Papaver bracteatum*	1X	KSCCKSTACRLKFPRKTCASTCNCKILKGNKCPSGYPK	1	9.72	4155.03
PcTHI-FYSJ-17384*^8^	*Polygonum convolvulus*	1X	TVCCPSTATRNAYTVCVSGGLSSSDCALVCGCITQTSSTCESGYDY	1	4.03	4666.16
NaTHI-ZENX-85534*	*Neurachne alopecuroidea*	1X	KVCCKTEIDMYCYDVCHCLKRPDELCAPLCNCTISDICPPDHPK	1	5.40	5017.93
		**7**			**8.32**	**4608.0**
SvTHI-EDIT-887*	*Sesuvium ventricosum*	2X	RICCRTPTAKTIYNTCRNAGGSRQMCAKLSGCHIVVGPCPVGCNHVSLQ	3	9.27	5206.14
SvTHI-HZTS-5985*	*Sesuvium portulacastrum*	2X	KSCCPFAGGSRPMCASLSGCKIVNGNKCPPGYPH	1	9.04	3468.08
SvTHI-OPZX-76	*Sesuvium ventricosum*	2X	SISCPSRSARNIYNVCRLGGASKNTCAQLSGCTVTSVS	4	9.30	3892.41
SvTHI-ZJDK-458*	*Sesuvium ventricosum*	2X	KSCCPTITARNIYNACRVPGTPRPVCASLNEYCKLGCVSSVCGAMVTL	1	8.82	5052.00
ChTHI-GJNX-209	*Cypselea humifusum*	2X	VLISLMMSQFGVEGISCPSASAKNIYIICRVAGGSESACSKLSGCTLTLGK	1	8.50	5183.13
ZpTHI-BERS-225	*Zaleya pentandra*	2X	QIQVEANIYNTCRFAGGSRDQCAKLSGCKHMDTCVPPYTK	1	8.49	4435.06
AlTHI-EMIG-63*^8^^,^^15^	*Aerva lanata*	2X	RTKCCCPDYIATNKYKVCRFAGASRAQCASLTGCIHITESTCPSSNPI	6	8.57	4913.69
BYNZ-2059306*^8^^,^^15^ BLWH-2085188*	*Portulaca mauii*	2x	RTKCCCPDYIATNKYKVCRFAGASRAQCASLTGCIHITESTCPSSNPI	2	8.81	5170.98
NaTHI-ZENX-85558	*Neurachne alopecuroidea*	2X	KSCCCSTTARNIYNGCRAAGFSREKCASVSGCKIVEGKCKPPCDRFSDS	1	8.91	5225.99
BnTHI-IPWB-14968*	*Brassica nigra*	2X	RVCCPSLSASNVFMVCCIQFPRSACLSVSGCIQVSGNVCPSGYSN	1	8.28	4675.46
TpTHI-QNIK-96290	*Trianthema portulacastrum*	2X	KSCCPSTTARNIYNSCRFTGGSRDMCASLSGCKIVNGSKCPSCCPKLAID	1	8.92	5236.08
		**11**		**225**	**8.81**	**4769.0**
		**133**				

For comparison, some typical previously known thionins have been included. Shown are the thionin name, plant species, class affiliation, sequence, number of hits, pI and molecular weight (Mw). Cysteine yellow, basic amino acids blue, acidic amino acids red, tyrosine pink, phenylalanine green.

* uneven number of cysteine residues,

^1^HTH1,

^2^Crambin,

^3^ViscotoxinA3,

^4^pTTH20,

^5^Same sequence, unusual pro-domains!,

^6^Not complete,

^7^Same sequence,

^8^Signal peptide cleavage site unclear,

^9^Same sequence,

^10^Same sequence,

^11^Same sequence,

^12^Same sequence,

^13^Genomic sequence,

^14^Same sequence,

^15^Same sequence.

No thionin sequences were found in the transcriptomes of algae and mosses. The phylogenetically oldest hits were a class 1 thionin sequence from *Selaginella wallacei* and from *Selaginella stauntoniana* (phylum *Lycophyta*) and two class 2 thionin sequences from the fern *Cystopteris fragilis*, indicating that thionins have perhaps evolved in the first vascular plants. Furthermore, class 1 thionin sequences were found in the conifers *Manoao colensoi*, *Sequoiadendron giganteum* and *Athrotaxis cupressoides* (two hits). Already these phylogenetically oldest thionins were produced as preproproteins including a typical pro-domain (“acidic” domain).

The 1KP project had reported that some data have to be treated with caution (https://cyverse.atlassian.net/wiki/spaces/iptol/pages/242170988/Sample+source+and+purity), including the *Cystopteris fragilis* XXHP sample that was flagged as “Worrisome Contamination”. The two hits for *Cystopteris fragilis* were from fronds (sample XXHP). A separate tissue sample from young *Cystopteris fragilis* leaves (not reported to be problematic) contained no hits. The 1KP project had also sequenced the transcriptomes of three other *Cystopteris* species and these contained also no hits for thionins. We tried to confirm the thionin sequences in the genome of *Cystopteris fragilis* by PCR but were unable to find these sequences. We must therefore conclude that the thionin sequences from *Cystopteris fragilis* were probably a contamination. In case of the *Selaginella* thionins, the hits were from two different species (in total, transcriptomes of 8 species were sequenced). In this case we had no plant material available to confirm the sequence by PCR.

**Fig 2:**
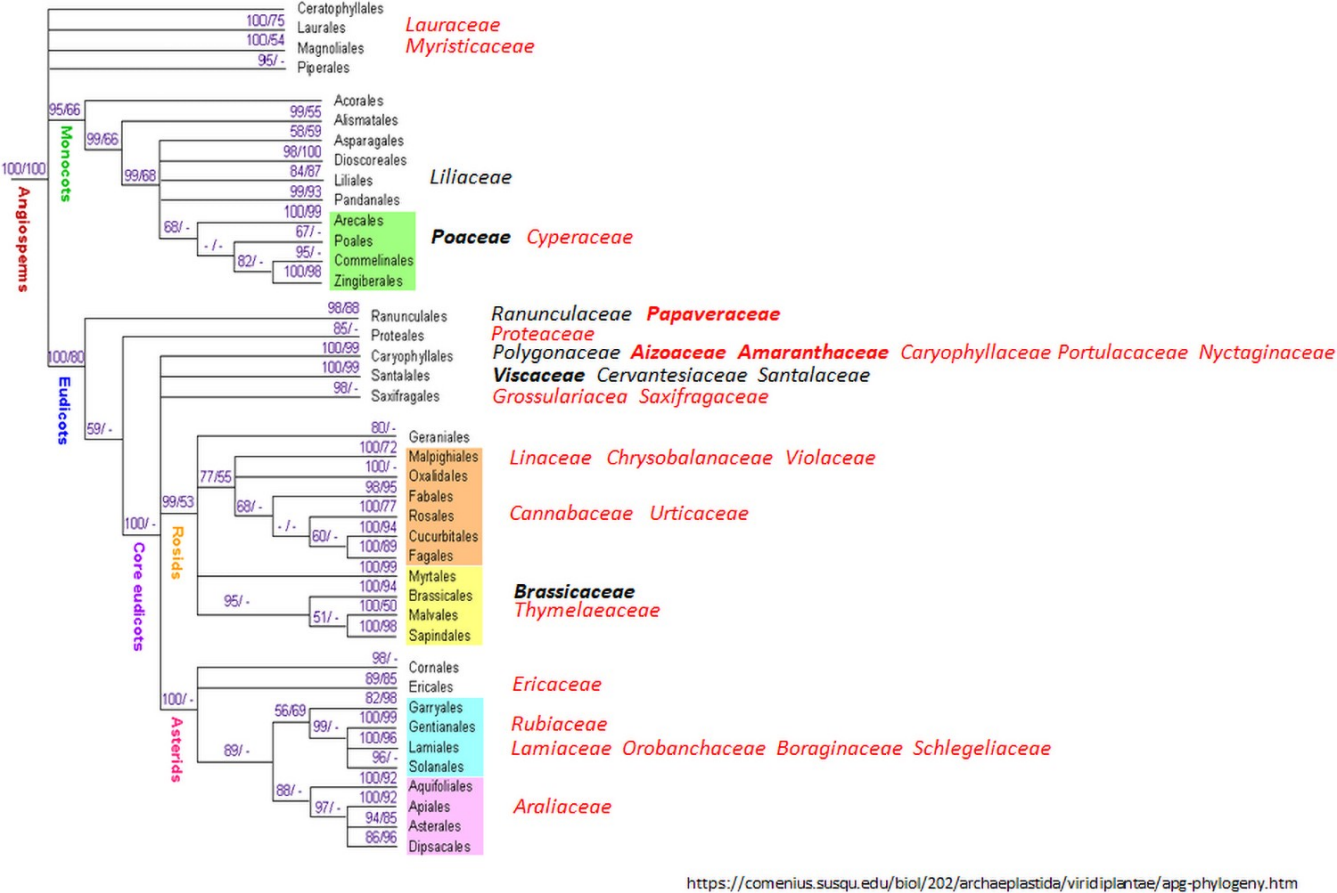
Phylogeny of angiosperms showing plant families with species having thionins. Black, families containing previously known thionins; red, families with newly found thionins from the 1Kb project. Bold, families containing many thionins. Phylogeny modified from https://comenius.susqu.edu/biol/202/archaeplastida/viridiplantae/apg-phylogeny.htm.

**Fig 3:**
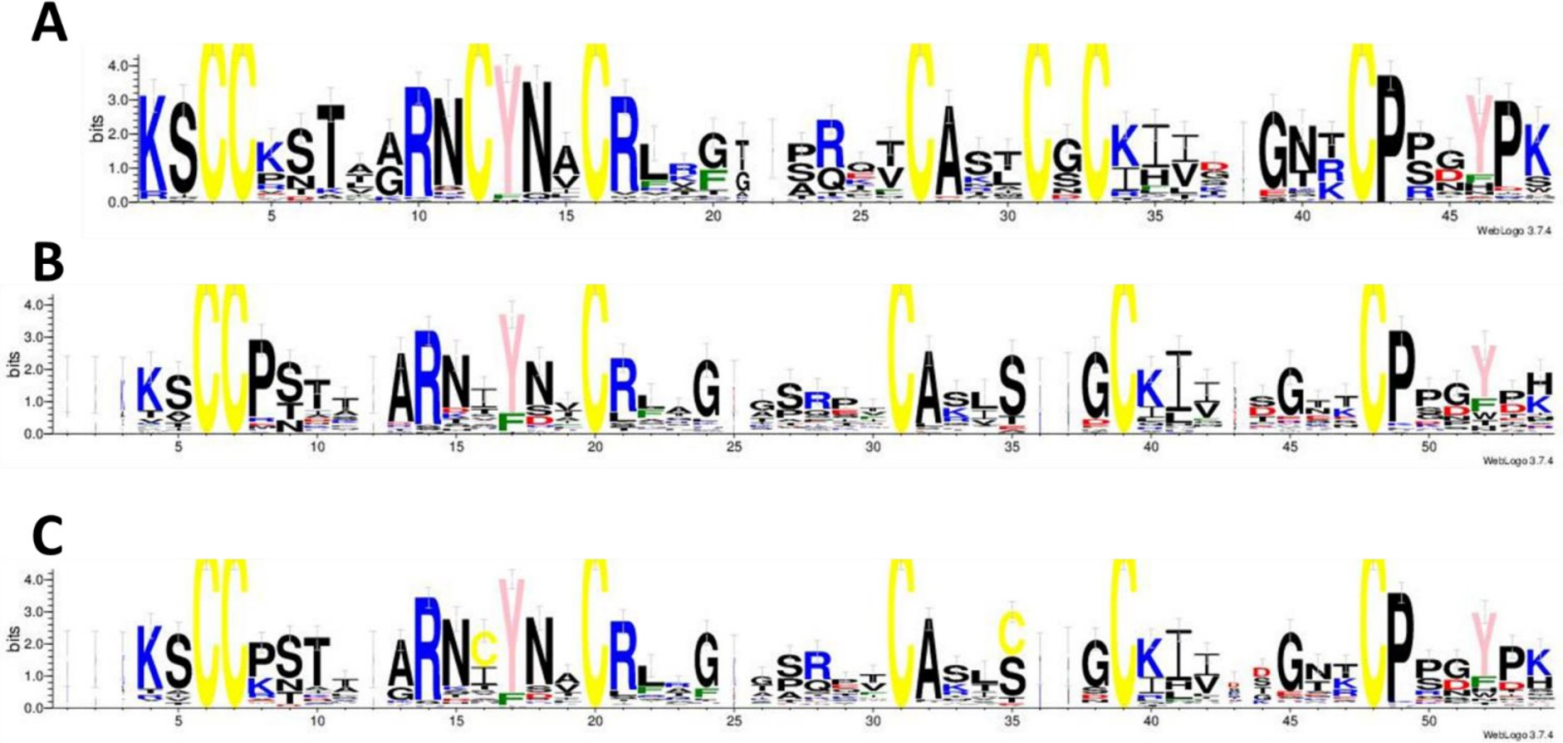
Sequence logos for thionins discovered by the 1KP project. A, class 1 thionins; B, class 2 thionins; C, class 1 and class 2 thionins. Cysteine yellow, basic amino acids blue, acidic amino acids red, tyrosine pink, phenylalanine green.

In total, 53 different class 1 sequences and 62 class 2 sequences were found, many of them in plant families of angiosperms from which previously no thionins had been reported (Fig 2). These class 1 and class 2 thionins had an average molecular weight of 4939.41 and 4959.49 Da, respectively. In general, there was more variability within the class 2 thionins than within the class 1 thionins (Table 2 and Fig 3). Of the 50 class 1 sequences that were not truncated at the N-terminus, four started with arginin as the first amino acid and one with isoleucine. All others had lysin as the first amino acid. In case of the class 2 thionins only 40 of 59 N-terminally untruncated sequences had lysin as first amino acid. In addition, seven sequences had an additional amino acid at the N-terminus, thus the double cysteine was at position 4/5 instead of 3/4 as determined by SignalP. One thionin had a N-terminal sequence of KSKKACC.

The average pI was 9.18 and 8.47 for class 1 and class 2 thionins, respectively. Thus, the class 1 thionins are more basic with no member having a pI below 7 while there were eight class two members with an acidic pI. The lowest pI of 4.04 was found for AaTHI2.3 from *Arabis alpina*. There were two more thionins from this species with an acidic pI while three had a basic pI. All *A*. *alpina* hits were from young leaves. The most basic thionin with a pI of 11.17 was also a class 2 thionin (DsTHI2.1 from *Draba sachalinensis*) with nine basic residues and no acidic residue. In addition to class 1 and class 2 thionins, variants were discovered that were previously unknown and which could have been evolved from class 1 (seven) or class 2 [11] thionins by deletion or insertion of cysteine residues. Some of these variants had an uneven number of cysteine residues (Table 2).

### A large gene family for thionins in *Papaver* species

**Fig 4:**
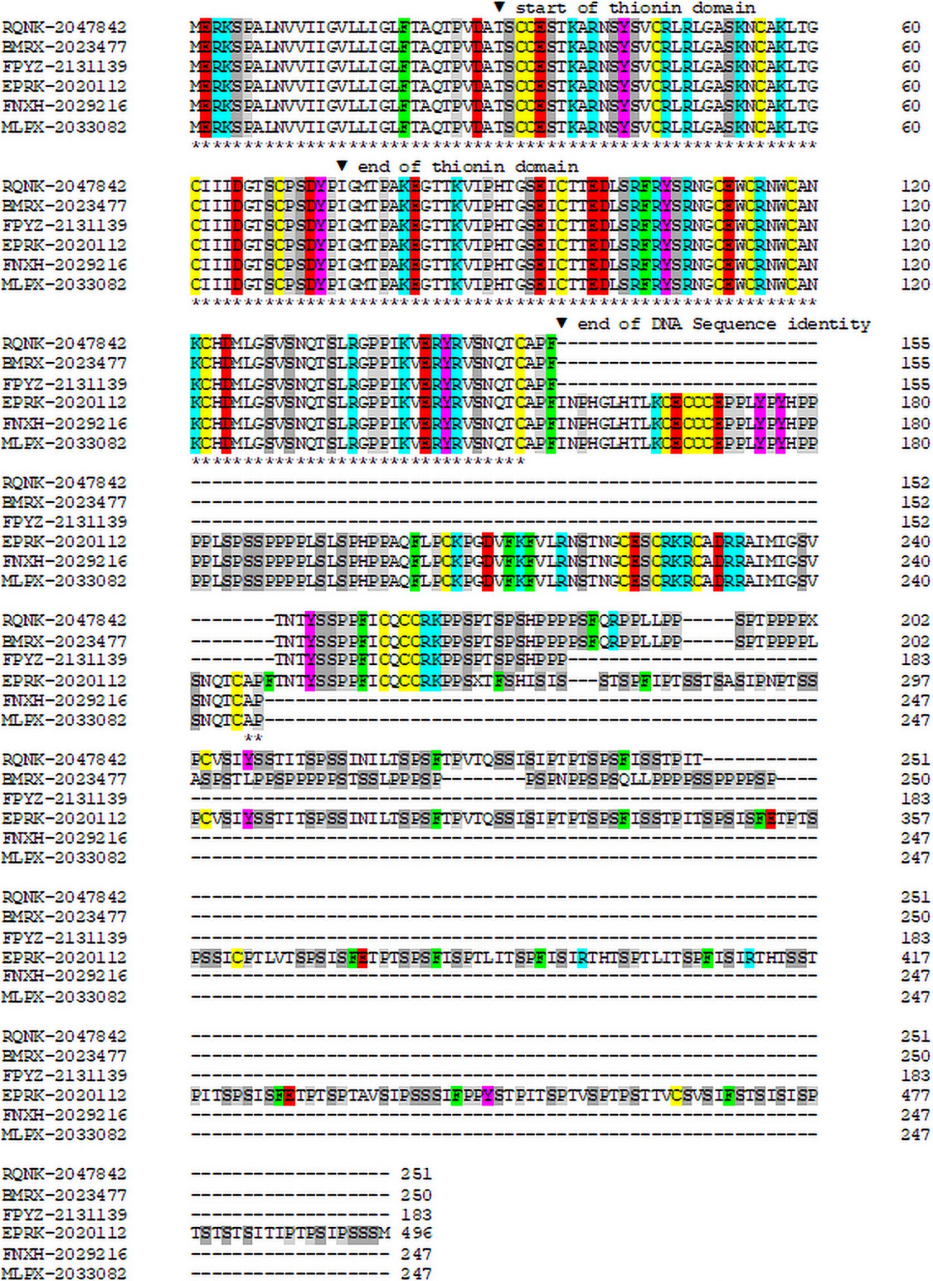
Alignment of *Papaver* thionin preproproteins with unusual pro domains. RQNK-2047842, BMRX-2023477 and FPYZ-2131139 are from *P*. *somniferum* (PsoTHI2.1) while EPRK-2020112, FNXH-2029216 and MLPX-2033082 are from *P*. *setigerum* (PseTHI2.1). Cysteine yellow, basic amino acids blue, acidic amino acids red, tyrosine pink, phenylalanine green, proline grey, serine dark grey.

Our results revealed a large gene family for thionins in *Papaver* species. Different tissues of several *Papaver* species resulted in a total of 93 hits. We found 11 class 1 thionins for *P*. *rhoeas*, six for *P*. *somniferum*, three for *P*. *setigerum* and seven for *P*. *bracteatum* (Table 2). In addition to class 1 thionins, all four *Papaver* species contained a few class 2 thionins, two in *P*. *rhoeas* and one each in the other three species. While the majority of *Papaver* thionins had the usual acidic domain, there were a few class 1 thionins from *P*. *somniferum* and *P*. *setigerum* which had an unusual pro domain (Fig 4). We found three hits each in both species. These were from developing fruits, leaves and stems in case of *P*. *setigerum* while the *P somniferum* hits were from flower buds, leaves and “five samples combined”. Following the thionin domain (pI 8.63) was a sequence with six cysteine residues and a pI of 8.89. Up to there all sequences were identical, which holds also for the DNA sequences. Following this were extensions with several cysteine residues which were rich in proline and serine residues. However, they were all truncated with the exception of EPRK-20112 from *P*. *setigerum* with a total of 496 amino acids. This extension contained 21 cysteine residues and was also basic with a pI of 8.53.

All *Papaver* species contained in addition variants (Table 2) of class 1 thionins with most of them having an uneven number of cysteine residues. We found variants with seven cysteines in *P*. *rhoeas*, *P*. *somniferum and P*. *bracteatum*. *P*. *setigerum* had one variant with nine cysteines. One *P*. *setigerum* thionin contained six cysteine residues, thus, seemingly being a class 2 member. However, closer inspection revealed a deletion of two cysteines from a class 1 thionin.

**Fig 5:**
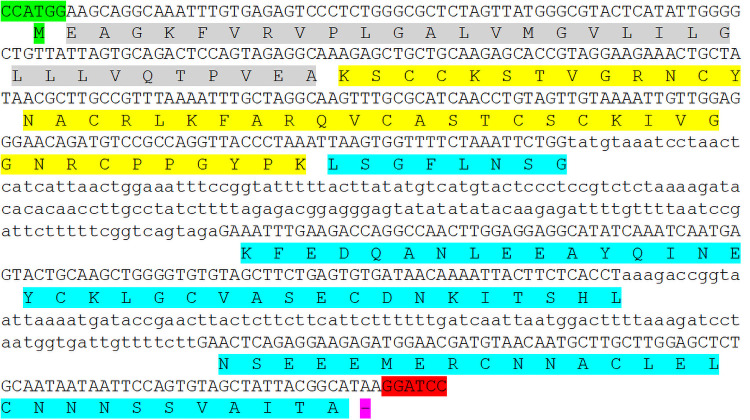
PsoTHI1.7 encoded by the genomic clone from *Papaver somniferum*. Capital letters are exons while lowercase letters indicate introns. Signal peptide marked grey, thionin domain marked yellow and acidic domain marked cyan. NcoI site marked green and BamHI site marked red.

From other thionin genes it is known that the acidic domain contains two small introns (S3 Fig). The transcriptome data from the 1KP project give of course no indication about introns. We have therefore cloned a genomic sequence from *Papaver somniferum* as described in Materials and methods. We derived the primer sequences from the DNA sequence of the *P*. *somniferum* clone BMRX-2017616 which encodes PsoTHI1.5. The genomic clone that we obtained coded for a typical thionin preprothionin and contained two small introns within the region of the acidic domain (Fig 5). The thionin sequence had two mismatches with PsoTHI1.5 and one with PsoTHI1.6 (Table 2) and was named PsoTHI1.7. It has a pI of 9.54 and a Mw of 4859.75 while the acidic domain has a pI of 4.26 and a Mw of 7724.47. The PsoTHI1.7 pI is similar to that of the other class 1 *Papaver* thionins (Table 2).

**Fig 6:**
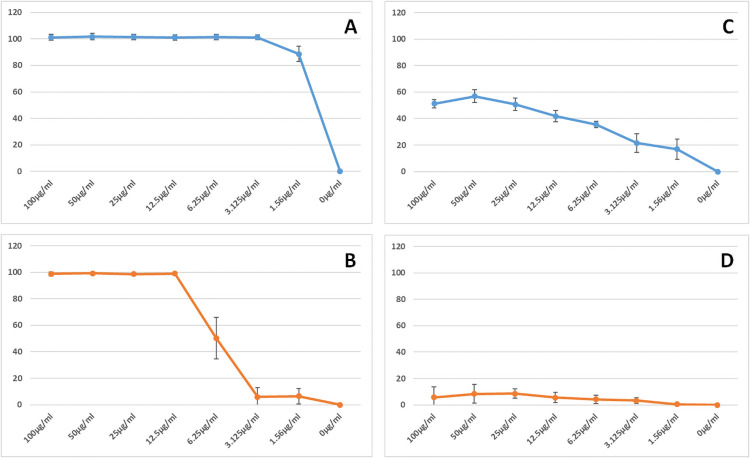
PsoTHI1.7 antimicrobial activity. A, *Fusarium oxysporum*; B, *Botrytis cinerea*. Growth inhibition was calculated after growth for 48h. Values are from 3 biological replicates with standard deviation. C, *Pseudomonas syringae*; D, *Agrobacterium tumefaciens*. Growth inhibition was calculated after growth for 24h. Values are from 3 biological replicates with standard deviation.

**Fig 7:**
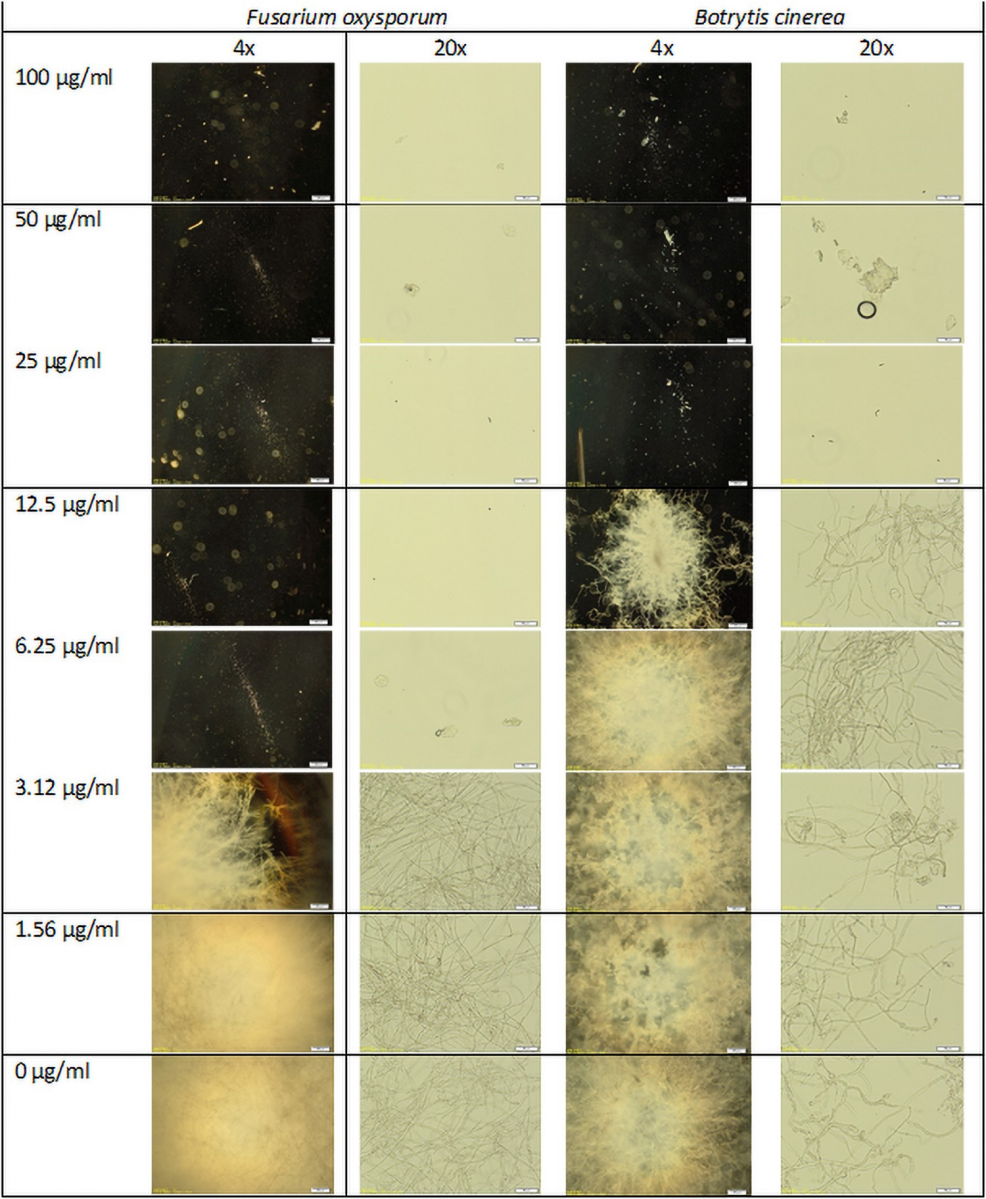
Representative pictures taken from the microtiter plate wells of antimicrobial assays for *B*. *cinerea* and *F*. *oxysporum*.

Basic thionins have repeatedly been reported to have antimicrobial activity. Having cloned the PsoTHI1.7 we used this sequence to produce an expression vector for *E*. *coli*. We produced PsoTHI1.7 as a fusion protein with thioredoxin, purified the fusion protein and cleaved it with TEV protease. Finally, PsoTHI1.7 was purified as described in Methods. MALDI-TOF mass spectroscopy showed that the size of the purified peptide corresponded to an oxidized peptide with all four disulfide bonds. We tested the antimicrobial activity against *Fusarium oxysporum* and *Botrytis cinerea* (Figs 6 and 7). The IC_50_ was approximately 1 μg/ml for *F*. *oxysporum* and 6 μg/ml for *B*. *cinerea* after 48 hours. Microscopic inspection of the hyphae revealed no morphological alterations such as hyperbranching or swelling of hyphal tips (Fig 7). The activity against bacteria was much lower. PsoTHI1.7 was not active against *Agrobacterium tumefaciens* but had some activity against *Pseudomonas syringae* with an IC_50_ of approximately 25 μg/ml (Fig 6).

### Variants

In addition to the thionin variants found in *Papaver* species, variants were in addition found in a number of species from the order *Caryophyllales*. *Sesuvium ventricosum* (Fam. *Aizoaceae*, order *Caryophyllales*) had a typical class 2 thionin and in addition three different class 2 variants with a typical acidic domain. Two of them had an uneven number of cysteines resulting from an additional residue. The other one had only four cysteine residues, missing cysteine number one plus another cysteine residue which was hard to define. This thionin was rather small with only 28 amino acids and was basic with a typical acidic domain. A second *Sesuvium* species, *S*. *portulacastrum*, contained another class 2 variant with a total of only 34 amino acids, this time with five cysteine residues but also with a typical acidic domain. The class 2 variant from *Cypselea humifusum* (Fam. *Aizoaceae)* had a typical acidic domain but was probably truncated at the N-terminus as SignalP did not find a signal peptide.

Several other species of the *Caryophyllales* contained also class 2 variants. *Aerva lanata* and *Portulaca mauii* both contained a variant with three consecutive cysteines at position 4–6 resulting in an uneven number of cysteines. Another class 2 variant with three consecutive cysteines, this time at position 3–5, was found in *Neurachne alopecuroidea*, a perennial grass. This variant has another additional cysteine residue, resulting in an even number of eight cysteines. *N*. *alopecuroidea* is a species with class 1 and 2 thionins and which contains in addition variants of class 1 (with an uneven number of cysteines due to an additional residue) and the already mentioned variant of class 2.

## Discussion

Thionins have originally been isolated as peptides from a number of plant species, especially mistletoes and cereals. Later on, DNA sequences have revealed thionin sequences in various other plant species. However, compared to defensins, there were many plant families for which no thionins had been reported. The 1KP project was thus an opportunity to extend the knowledge about the distribution of thionins within plants. Since the 1KP project also sequenced a number of algae, mosses, ferns and conifers it was also possible to test if thionins might be found in plant species other than angiosperms. Using various bait sequences we found 225 thionins. The 1KP project sequenced cDNAs from specific plant organs but also pooled samples which resulted in multiple hits for some thionins. In addition, some thionins were found in different samples. This reduced the number to a total of 133 different thionins.

We did not find thionin sequences in algae and mosses. Shelenkov et al. [64] also recently mined the 1KP data for AMP sequences, including thionins. They reported that they found thionin sequences in algae with 36 thionins in *Chlamydomonadaceae* and 10 in *Desmidiaceae*. We had 31 primary hits in each of these families. However, thorough inspection showed that all these hits did not contain thionins. Since Shelenkov et al. [64] reported only numbers without sequences it is at the moment not possible to resolve these differences.

The phylogenetically earliest hits were from *Selaginella wallacei* and *S*. *stauntoniana* which belonged to class 1. We had no plant material available to confirm the sequences by PCR. We found four class 1 sequences from conifers, one from *Manoao colensoi* (Fam. *Podocarpaceae*) and the same sequence with two hits from *Athrotaxis cupressoides* (Fam. *Cupressaceae*). Furthermore, another species from the family *Cupressaceae*, *Sequoiadendron giganteum*, also had one hit. All other sequences were from angiosperms. Up to the 1KP project there were eight plant families of the angiosperma which were known to contain thionins. The 1KP project identified 25 plant families which contained previously no known thionins. However, there are still many plant families which do not seem to contain thionins, although the negative results from the 1KP project have to be treated with care. All these data are from transcriptomes, sometimes only from one tissue. It is therefore possible that genes which are not expressed in that tissue or only expressed at a low level are missed. In those cases the genome sequence is needed.

According to the data from the 1KP project, thionins emerged first in lycophytes as class 1 thionins and class 1 thionins are also found in conifers. Class 2 thionins then came up within the angiosperms which now contain both classes. Most plant families contain only one class of thionins. However, there are also plant species which contain both classes of thionins. *Poaceae* have mainly class 1 thionins while *Brassicaceae* have mainly class 2 thionins but there are some exceptions. *N*. *alopecuroidea*, family *Poaceae*, had a class 1 and a class 2 thionin. The majority of thionins in the *Papaver* species are class 1 but all four species that were included in the 1KP project also contain one class 2 thionin (two in the case of *P*. *rhoeas*).

There was a slightly higher number of class 2 thionins than class 1 and half of the class 1 thionins were from *Papaver* species. This might indicate that class 2 thionins are perhaps more widely distributed than class 1 thionins. In line with this, the variability among class 2 thionins was higher and acidic thionins were only found within class 2.

### Large gene family for thionins in *Papaver* species

The 1KP project revealed a large number of thionin sequences in all four *Papaver* species that were sequenced. It was previously not known that *Papaver* species contain thionins, supporting the importance of large scale transcriptome and genome projects. The *Papaver* thionins included typical class 1 and class 2 thionins as well as several variants (see below). In addition, class 1 thionins from *P*. *somniferum* and *P*. *setigerum* were discovered which had an unusual pro domain. Most of the clones were truncated but one seemed to be complete with a total of 496 amino acids. It is unlikely that these clones were artifacts because they were found in 3 samples each of 2 different *Papaver* species. However, it would still be important to confirm these with genomic sequences. Furthermore, this unusual structure also raises the question how the pro domain is processed. Could the processing give rise to not only a thionin but also additional protein/s with specific functions? This should be investigated experimentally.

We isolated a genomic clone from *P*. *somniferum* which allowed us to check for introns because this information is of course not evident from transcriptomiocs. We found two small introns within the region encoding the acidic domain. These two small introns seem to be generally conserved in thionin genes [19,42,47]. It would be interesting to see if these are already found in the phylogenetically earliest thionin genes.

We expressed the *Papaver* thionin PsoTHI1.7 in *E*. *coli*. It had significant antimicrobial activity *in vitro* against the fungi we tested but only low activity against *P*. *syringae*. This is in line with other thionins that have been tested *in vitro*, for instance TaTHI1.3, α-purothionin [65]. Molina et al. [33] also tested wheat purothionins against different pathogens. For three different *B*. *cinerea* strains they found IC_50_ values between 5 and 12 μg/ml which is comparable to the IC_50_ value of 6 μg/ml for the *B*. *cinerea* strain that we tested. All *Papaver* class 1 thionins have a bacic pI and contain lysin as first amino acid. Most of them also have tyrosin at position 13, indicating that these have most likely antimicrobial or toxic activities [4]. If we compare PsoTHI1.7 with the other *Papaver* class 1 thionins it is safe to conclude that also the other class 1 thionins will have antimicrobial activity, maybe with the exception of those that do not have tyrosin at position 13. With all the data available, it is very likely that the *Papaver* thionin gene family is involved in resistance against pathogens.

### Variants and a new classification for thionins

Thionins were previously grouped into four classes [18]. With the data obtained by the 1KP project we will now recognize only two classes and variants thereof. The reason for this is that all the variants that we found can be traced back to either class 1 (eight cysteines) or class 2 (six cysteines). With all the different variants (a total of 18 variants) uncovered by the 1KP project we would have to extend the number of classes substancially, making the system confusing. Accordingly, TaTHI3.1 and TgTHI4.1, formerly class 3 and class 4 thionins [18], respectively, are both variants of class 1. Many of the variants that we found here, six of the seven class 1 variants and six of the 11 class 2 variants, have an uneven number of cysteine residues. This raises the question about the structure of the peptides. We can assume that most of these cysteines form disulfide bridges, as has repeatedly been shown for many class 1 and class 2 thionins, leaving probably one single cysteine. It could be possible that the single cysteines engage in forming dimers. It has been shown before that thionins without single cysteines form dimers [44]. However, formation of intermolecular disulfide bridges has to be determined experimentally by isolating the peptides from the plants or by expressing them in expression hosts.

## Supporting information

S1 Fig Sequences of different thionin proproteins. (DOCX)

S2 Fig General structure of thionin preproproteins and position of the introns. (DOCX)

S3 Fig Expression and purification of PsoTHI1.7. (DOCX)

S1 Table pI of some thionins, acidic domains and proproteins. (DOCX)

S2 Table Bait sequences that were used to search the 1KP data. (DOCX)

S3 Table Primers used in this work. (DOCX)
