# Supplementary material for: The thionin family of antimicrobial peptides
Source: PLoS One. 2021 Jul 14;16(7):e0254549. doi: 10.1371/journal.pone.0254549 (PMC8279376; doi:10.1371/journal.pone.0254549)
Supplement: S1 Fig — (DOCX) [file pone.0254549.s001.docx]

**Figure S1. Sequences of different thionin proproteins**

**TaTHI1.3 KSCCKSTLGRNCYNLCRARGAQK-LCANVCRCKLTSGLSCPKDFPK▼**

**HvTHI1.1 KSCCRSTLGRNCYNLCRVRGAQK-LCAGVCRCKLTSSGKCPTGFPK▼**

**BTH6** **KSCCKDTLARNCYNTCRFAGGSRPVCAGACRCKIISGPKCPSDYPK▼**

**AtTHI2.1 KICCPSNQARNGYSVCRIRFSKG-RCMQVSGCQNS--DTCPRGWVN▼**

**AtTHI2.2 KICCPTKDDRSVYFVCMLSVSSQFYCLLKSKCKNTSQTICPPGYTN▼**

**AtTHI2.3 KTCCPSQSTRKEFEDCISEGNLQILCSAESGCRDTYVGYCPSGFPY▼**

**AtTHI2.4 NICCPSIQARTFYNACLFAVGSPSSCIRNSSCLDISESTCPRGYTN▼**

**VaTHI2.1 KSCCPNTTGRNIYNACRLTGAPRPTCAKLSGCKIISGSTCPSDYPK▼**

**LVLESNSDEPDTMEYCNLGCRSSLCDYIVNAAA-DDEEMKLYV-EQCGDACVNFCNADAGLTSLDA**

**LALVSNSDEPDTVKYCNLGCRASMCDYMVNAAA-DDEEMKLYL-ENCGDACVNFCNGDAGLTSLTA**

**LNLLPESGEPDVTQYCTIGCTNSVCDNMDNVFR--GQEMKFDMG-LCSNACARFCNDGAVIQSVEA**

**AILE-NSADA--NEHCKLGCETSVCGAMNTLQNSDASEIVNGASEQCAKGCSIFCTKSYVVPPGPPKLL**

**DILE-NSGDA-VNEYCKLGCASSVCGALTTLQNFDTSKVLSEAVEQCTKACSSVCTGGSTAAVKSA**

**GSLT-NSGDV-VNVYCKLGCVSSLCGALTSLQKLDTSGKVNVAVERCTKACSTICTKGSKTAVETV**

**DILE-NTGDA-VTEYCKLGCVSSVCGALTILQNSDASEIVNGEVEKCTMACSTVCTKGSMNAVENA**

**-------------FYCTMGCESSQCATNS---NGDA-EAV-----RCKTACSDLCQ-----DVDDA**

Basic amino acids marked blue, acidic amino acids red, cysteine yellow, tyrosin lilac, phenylalanine and tryptophan green. **▼** Indicates processing site between thionin and acidic domain.
