# Supplementary material for: The thionin family of antimicrobial peptides
Source: PLoS One. 2021 Jul 14;16(7):e0254549. doi: 10.1371/journal.pone.0254549 (PMC8279376; doi:10.1371/journal.pone.0254549)
Supplement: S2 Fig — (DOCX) [file pone.0254549.s002.docx]

**Figure S2. General structure of thionin preproproteins and position of the introns.**


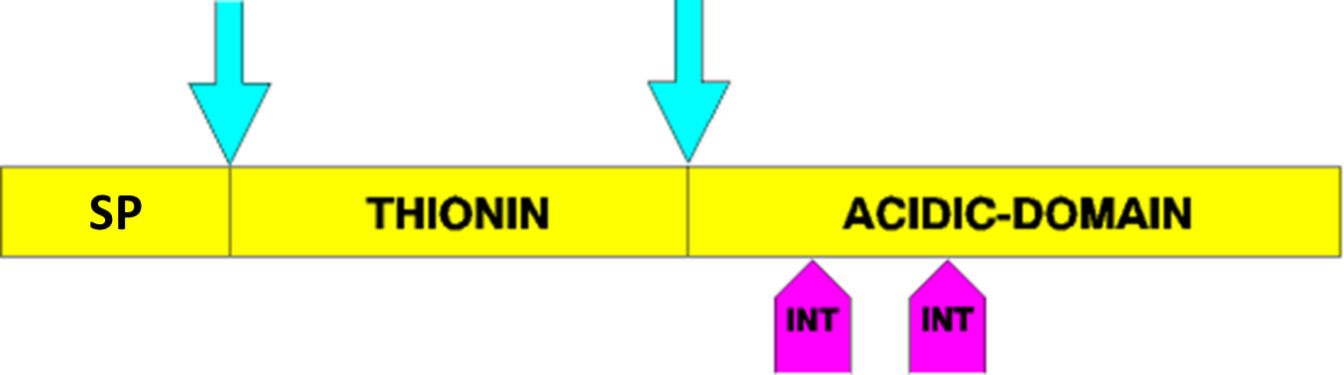


Thionin preproproteins consist of a signal peptide, the thionin domain and the acidic domain. The acidic domain usually contains 2 small introns. Arrows indicate the processing steps between signal peptide and thionin and between thionin and acidic domain, respectively, that are necessary to produce the mature thionin. SP, signal peptide; INT, intron.
