# Supplementary material for: The thionin family of antimicrobial peptides
Source: PLoS One. 2021 Jul 14;16(7):e0254549. doi: 10.1371/journal.pone.0254549 (PMC8279376; doi:10.1371/journal.pone.0254549)
Supplement: S3 Fig — (DOCX) [file pone.0254549.s003.docx]

**Figure S3. Expression and purification of PsoTHI1.7.**


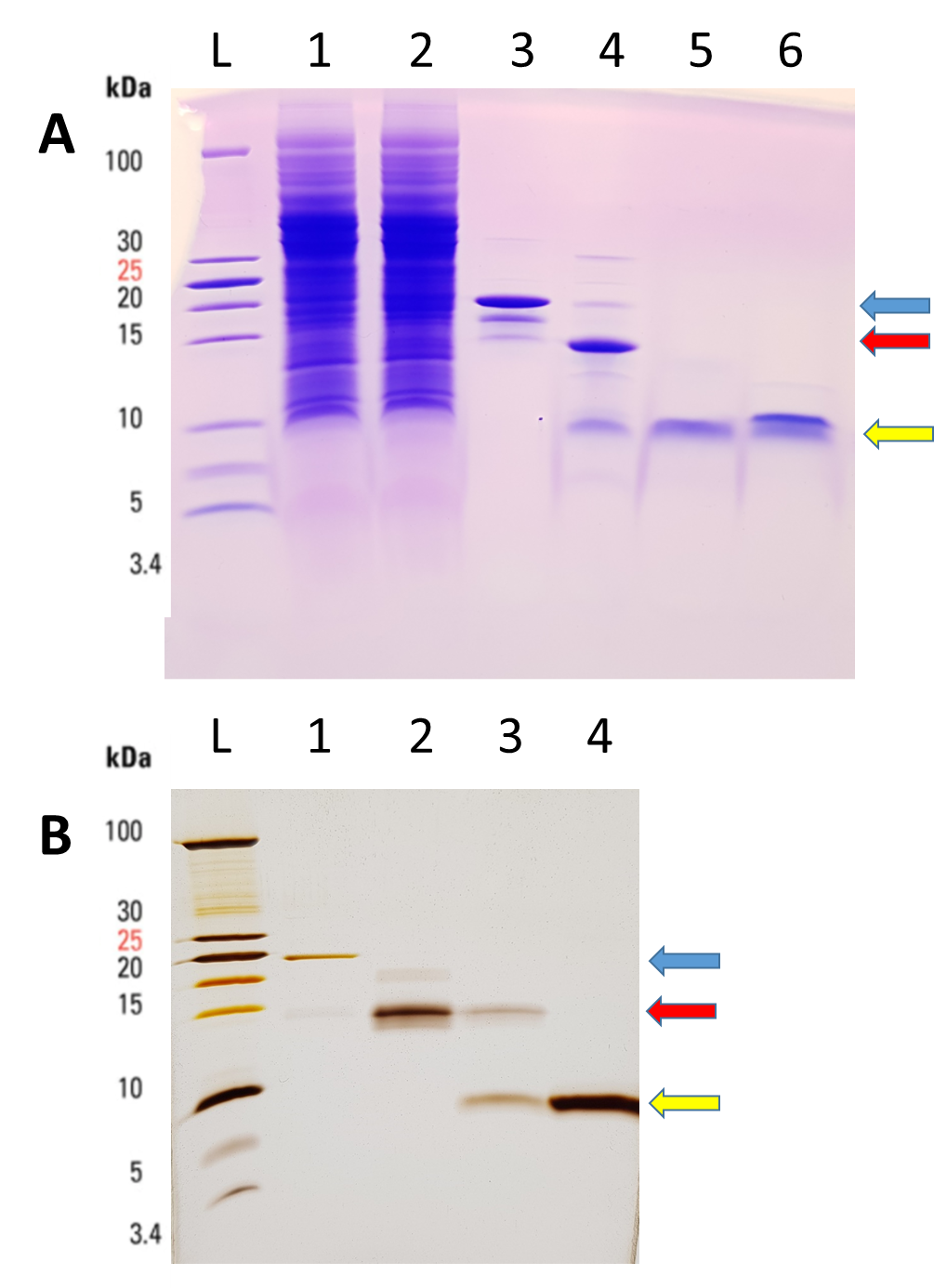


A; L Ladder, 1 non induced, 2 induced, 3 Histag purification, 4 TEV digestion, 5 flow-through of (negative) Histag purification #2, 6 Gel filtration.

B; Purification with gel filtration: L Ladder, 1 peak 1 (A1) = remaining fusion protein, 2 peak 2 (A3) = TRX, 3 peak 3 (A4+A5) = TRX and some psThi, 4 peak 4 (A6) = PsoTHI1.7 running as dimer

Blue arrow, fusion protein; red arrow, TRX; yellow arrow, thionin
