# Supplementary material for: The thionin family of antimicrobial peptides
Source: PLoS One. 2021 Jul 14;16(7):e0254549. doi: 10.1371/journal.pone.0254549 (PMC8279376; doi:10.1371/journal.pone.0254549)
Supplement: S1 Table — (DOCX) [file pone.0254549.s004.docx]

**Table S1** isoelectric points

| Name | Original Name | Thionin | Acidic domain | Proprotein |
| --- | --- | --- | --- | --- |
| CaTHI2.1 | Crambin | 5.73 | 6.24 | 6.00 |
| HvTHI1.1 | Hordothionin α | 9.75 | 3.82 | 7.96 |
| HvTHI1.3 | DB4 | 9.06 | 4.16 | 7.55 |
| AtTHI2.1 |  | 9.43 | 4.75 | 8.30 |
| AtTHI2.2 |  | 8.86 | 4.44 | 7.56 |
| AtTHI2.3 |  | 4.66 | 8.63 | 7.56 |
| AtTHI2.4 |  | 7.77 | 4.06 | 4.41 |
| VaTHI2.1 | Viscotoxin A3 | 9.30 | 3.85 | 7.57 |
