# Supplementary material for: The thionin family of antimicrobial peptides
Source: PLoS One. 2021 Jul 14;16(7):e0254549. doi: 10.1371/journal.pone.0254549 (PMC8279376; doi:10.1371/journal.pone.0254549)
Supplement: S2 Table — (DOCX) [file pone.0254549.s005.docx]

**Table S2**

Bait sequences that were used to search the 1KP data

| Name | Sequence |
| --- | --- |
| THI2.1 | KICCPSNQARNGYSVCRIRFSKGRCMQVSGCQNSDTCPRGWVN |
| VaTHI2.1 (Viscotoxin A3) | KSCCPNTTGRNIYNACRLTGAPRPTCAKLSGCKIISGSTCPSDYPK |
| CaTHI2.1 (Crambin) | TTCCPSIVARSNFNVCRLPGTPEALCATYTGCIIIPGATCPGDYAN |
| HvTHI1.3 (DB4) | KSCCKDTLARNCYNTCHFAGGSRPVCAGACRCKIISGPKCPSDYPK |
| PpTHI1.1 (Pyrularia thionin) | KSCCRNTWARNCYNVCRLPGTISREICAKKCDCKIISGTTCPSDYPK |
| TgTHI4.1 | KSCFPSTAAKYCYNACRLPGCRPETICAARCGCKIISSGNCPPGYDYEN |
| Hellethionin D | KSCCRNTLARNCYNACRFTGGSQPTCGILCDCIHVTTTTCPSSHPS |
| Rheum thionin | VVCCPSVAARSRYNMCRMVPEKCASFAGCLLVIRKCPPGWDNFNA |
| AL3G34680 | RTCCPSQSSREEFEDCISQGNLHTVCGAGSGCLESYVGFCPSQYPY |
| TaTHI1.3 (Purothionin A1) | KSCCKSTLGRNCYNLCRARGAQKLCANVCRCKLTSGLSCPKDFPK |
| Wheat thionin pTTH20 | VDCGANPFKVACFNSCLLGPSTVFQCADFCACRLPAG |
| K3 (*Brassica napus*) | KICCRNTRARNIFDSCRAQVGCTSSLCGPLTTVQNSGASEIGDGAVEQCANACSILCTTGSTKLAVETA |
| XXHP-9499-Cystopteris_fragilis | KTCCPSSTARSIYRTCRFGGSTQTCAQISGCKIVSGECPGGYNK |
| JKAA-16990-Selaginella_wallacei | KSCCPSTAARNCYNACRLVGTSQTTCASLCGCIHVDGNTCPPNYPS |
| OBPL-2600-Myristica_fragrans | ESCCPSAKAKNLYNVCRNQYSDPHYFTKSFCANLAGCKLADGKKCEPPYDH |
| ICOX-50282-Eleusine_coracana | ISCCPDTTKRNCYNVCRHSMKKEICANVCGCKLVSGVKCPRDYPK |
