# Supplementary material for: The thionin family of antimicrobial peptides
Source: PLoS One. 2021 Jul 14;16(7):e0254549. doi: 10.1371/journal.pone.0254549 (PMC8279376; doi:10.1371/journal.pone.0254549)
Supplement: S3 Table — (DOCX) [file pone.0254549.s006.docx]

**Table S3**

Primers used in this work

| PsoThi1.5forNco | AAGACCATGGAAGCAGGCAAATTTGTG | Amplify thionin from Papaver somniferum |
| --- | --- | --- |
| PsoThi1.5revBam | AACGGATCCTTATGCCGTAATAGCTACAG |  |
| PsoThi1.5for | CTA AGA GCT GTT GCA AGA GCA CC-3' | Papaver somniferum Thi1.5 |
| PsoThi1.5rev | CGT CTC TTT ATG CCG TAA TAG CTA C-3' |  |
| TEV2PsoTH1.7for | AAAACTTGTACTTCCAGAAGAGCTGCTGCAAGAGCACC | Cloning Papaver Thionin as fusion |
| PsoTH1.7Bamrev | AAAGGATCCTTATTTAGGGTAACCTGGC |  |
| TEV1PsoTH1.7for | GAGAATCTTTATTTTCAGAAGAGCTGCTGCAAGAGCACC | Cloning Papaver Thionin as fusion with TRX |
| pETtrxfor1 | GTCCGGCGTAGAGGATCG | Amplification of TRX fusion part of vector pETtrx1a |
| pETtrxTEVrev | CTGAAAATAAAGATTCTCAGA |  |
